# Engraftment Syndrome After Second Autologous Stem Cell Transplantation for Myeloma: A Case Report

**DOI:** 10.1155/crh/2925166

**Published:** 2026-08-25

**Authors:** Konstantina Salveridou, Theodoros Tzamalis, Maika Klaiber-Hakimi, Sabine Haase, Stefan Diederich, Aristoteles Giagounidis

**Affiliations:** ^1^ Department of Oncology, Hematology and Palliative Care, VKKD, Association of Catholic Hospitals Duesseldorf, Marien Hospital Duesseldorf, Düsseldorf, Germany; ^2^ Department of Oncology, Hematology and Palliative Care, Rheinland Klinikum Neuss, Neuss, Germany; ^3^ Department of Diagnostic und Interventional Radiology, VKKD, Association of Catholic Hospitals Duesseldorf, Marien Hospital Duesseldorf, Düsseldorf, Germany

## Abstract

Engraftment syndrome (ES) is a rare but challenging posttransplant complication, presenting during or after absolute neutrophil recovery. It may easily be confused with febrile infectious complications since its main clinical features include fever, extensive skin rash, diarrhea, and atypical pulmonary infiltrates. Its pathophysiology and potential trigger factors remain not well understood. An immunosuppressive therapy with corticosteroids represents the standard treatment, and most cases present a rapid resolution of symptoms after treatment initiation. Still, in cases of severe presentation including periengraftment respiratory distress syndrome (PERDS), a mortality rate up to 21% has been described. In this case report, we report the case of a male patient after second autologous stem cell transplantation (SCT) with recurrent febrile episodes with no response to usual antibiotic regimens. Our patient developed diarrhea from Day 9 post‐SC, escalating to a full‐body rash and atypical pneumonia on and after Day 16. Our diagnosis was based on the Maiolino criteria, and a corticosteroid treatment was instituted. The symptoms ceased 2 days later, while we continued the treatment for a week. Physicians should be aware of this rare clinical entity, which can mimic other common posttransplant complications, hindering prompt diagnosis and treatment.

## 1. Introduction

Engraftment syndrome (ES) is a clinical entity presenting as a febrile posttransplant complication. It is mainly associated with autologous stem cell transplantation (SCT) with an incidence between 7% and 59% and usually presents with atypical rash, fever, diarrhea, and aseptic pneumonia [1]. Nevertheless, it can be challenging to differentiate it from posttransplantational infectious complications due to the diversity of the syndrome itself and the complexity of the post transplantation phase. Early diagnosis may be crucial, since in individual cases, ES can be fatal; however, in most cases, it resolves after steroid treatment. In this article, we present an interesting case of ES after tandem transplantation with rapid remission of symptoms after administration of corticosteroids.

## 2. Clinical Presentation

**FIGURE 1:**
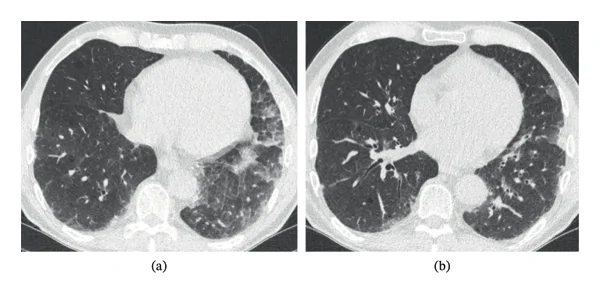
Unenhanced transverse thin‐section (0.625 mm) CT at lung windows with low‐dose settings. (a), (b) Subpleural ground glass, consolidation, and interlobular septal thickening with a basal predominance.

**FIGURE 2:**
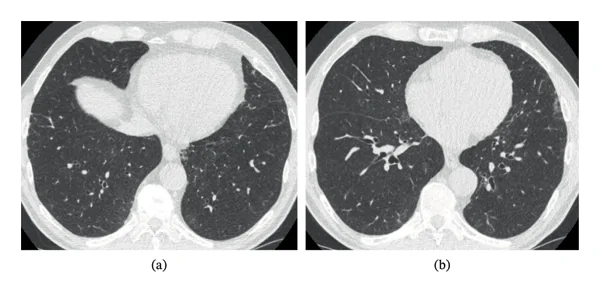
Unenhanced transverse thin‐section (0.625 mm) CT at lung windows with low‐dose settings. (a), (b) Follow‐up scan 20 days later: almost complete resolution of the abnormal findings.

A 62‐year‐old Caucasian male was admitted for a second autologous bone marrow transplantation to treat multiple myeloma. Four months before admission, he completed his first ASCT without complications. Our patient received a conditioning regimen consisting of melphalan (200 mg/m^2^). On day +4 post‐ASCT, he developed worsening diarrhea later accompanied with persistent fever and was empirically started on broad‐spectrum antibiotics with piperacillin/tazobactam. Blood and urine cultures were noncontributory. The transaminase and alanine concentrations were not elevated. Maximum axillary temperature was 39°C on day +9 post‐ASCT. CT examination of the lungs on that day revealed bilateral infiltrates (Figure 1). Clinical examination showed no respiratory constraint. There were no signs of kidney or liver function abnormalities. The patient was continued on piperacillin/tazobactam, and due to failure to respond, was switched to meropenem. The neutrophil count fully regenerated on Day 11 post‐ASCT. Physical examination revealed severe maculopapular exanthema on the trunk and back on Day 16 post‐ASCT. The exanthema extended to the extremities on Day 19 post‐ASCT. The diagnosis of ES was based on the Maiolino criteria [2]. We began a corticosteroid therapy with a dose of 1 mg/kg. Major diagnostic criteria of ES were body temperature of 39°C or more, maculopapular rash involving more than 20% of the body surface area, and pulmonary infiltrates. A skin or lung biopsy was not obtained at the patient’s request. The rash significantly abated after 2 days of steroid administration. We tailed off corticosteroid therapy within a week after symptom resolution. A CT scan 1 month after hospital discharge showed a complete remission of the early findings (Figure 2). Written consent was obtained from the patient (Table 1).

**TABLE 1:** Clinical and laboratory findings of the patient during the course of autologous stem cell transplantation.

Parameter	Finding	Time point/notes
Demographics	62‐year‐old Caucasian male	
Diagnosis	Multiple myeloma	Undergoing second ASCT
Conditioning regimen	Melphalan 200 mg/m^2^	Prior to second ASCT
Day +4 post‐ASCT	Diarrhea, persistent fever	Started empirical piperacillin/tazobactam
Blood and urine cultures CRP and procalcitonin	Negative to low levels	Throughout febrile period
Liver enzymes (AST/ALT)	Within normal limits	Throughout hospitalization
Maximum body temperature	39.0°C	Day +9 post‐ASCT
Chest CT	Bilateral pulmonary infiltrates	Day +9 post‐ASCT
Respiratory status	No respiratory distress	Despite CT findings
Kidney and liver function	Normal	Throughout course
Antibiotic therapy	Piperacillin/tazobactam switched to meropenem	Due to lack of clinical improvement
Neutrophil recovery	Full regeneration	Day +11 post‐ASCT
Skin findings	Severe maculopapular rash (> 20% BSA)	Day +16 post‐ASCT
Rash progression	Extension to extremities	Day +19 post‐ASCT
Diagnostic criteria for ES	Fever ≥ 39°C, maculopapular rash, pulmonary infiltrates	Met Maiolino criteria
Biopsy	Not performed	Patient declined
Treatment for ES	Corticosteroids (1 mg/kg prednisone equivalent)	Initiated post‐ES diagnosis
Clinical response to steroids	Rash improvement within 2 days	
Steroid tapering	Completed within 1 week postsymptom resolution	
Follow‐up chest CT	Complete resolution of infiltrates	1 month postdischarge

## 3. Discussion

ES presents at the phase of hematopoietic regeneration, usually occurring approximately between 10 and 20 days after autologous transplant. The first report of the syndrome has been attributed to Chang et al. in his retrospective analysis of 248 patients receiving ASCT. Fifty percent of the population developed a skin rash and noninfectious fever, capillary leak, pulmonary infiltration, or hypoxia with a median onset at 7 days post‐ASCT [3]. Katzel et al. reported an incidence of 10 ES cases out of 90 with multiple myeloma following ASCT and high‐dose chemotherapy [4]. More serious clinical courses with the development of full‐scale PERDS may occur in up to 5% of autologous transplants [5]. Our patient developed persistent fever, diarrhea, and bilateral pulmonary infiltrates on Day 9 posttransplantation, while the widespread rash appeared on Day 16, well in keeping with previous reports of ES. The diagnostic criteria include clinical features of the syndrome and are defined in detail by Spitzer, Maiolino, and others [1, 2]. The Spitzer criteria were established by reviewing the literature on ES‐like syndromes in patients after autologous, syngeneic, and allogeneic SCT. The Maiolino criteria were suggested based on a retrospective single‐center study of 125 patients following autologous transplantation. The main Spitzer criteria include fever (≥ 38.3°C), erythrodermatous rash over more than 25% of the body not attributable to medication, and noncardiogenic pulmonary edema, as well as a sudden increase in CRP values. The Maiolino criteria also include noninfectious fever and rash but include diarrhea or pulmonary infiltrates within 24 h of engraftment. Reviewing 178 patients after autologous HSCT, Sheth et al. reported that the Maiolino criteria had a higher sensitivity in their population [6]. Our case fulfilled all Maiolino criteria. Recurrent diarrhea in the context of an intestinal mucositis is rather common in neutropenic patients and thus tends to cloud the diagnostic landscape [7]. The pulmonary infiltrates depicted on the CT scan were reported as in keeping with atypical pneumonia of viral or mycotic origin. However, given the clinical suspicion of ES, we still proceeded with corticosteroid treatment that alleviated all symptoms within 48 h. The pathophysiology of engraftment remains unclear. An autoimmune inflammatory response seems to be responsible, since the onset of the symptoms coincides with the granulocytes’ regeneration, and there is a direct response to immunosuppressive therapy with corticosteroids. In recent literature, ES has been correlated with several parameters. Such parameters include granulocyte colony stimulation factors (G‐CSFs) administered after ASCT as well as the type of growth factor [8], specific hematological malignancies [9], type of previous chemotherapies [10, 11], and the cumulative exposure to them [12] An important pathogenetic mechanism seems to be the amount of proinflammatory cytokines released before and during neutrophil recovery. A significant higher level of IFN‐γ, TNF‐α, IL‐1β, IL‐6, and IL‐12 was detected in patients with ES compared to patients with more common complications after allogenic SCT like acute graft versus host disease. Such mechanisms, as well as T‐cell–mediated actions, may create an intolerance in the autologous setting [13]. In terms of prevention, there have been only isolated reports. A prophylactic therapy with the corticosteroid budesonide significantly reduced the likelihood of ES in patients with multiple myeloma undergoing ASCT. However, such therapy regimens lack supportive data from randomized studies [14]. Gimenez Zepeda et al. showed how the use of cyclophosphamide and prednisone induction chemotherapy followed by cyclophosphamide mobilization decreased the ES incidence after ASCT in patients with POEMS syndrome [11]. Interestingly, it appears that there is no difference in relapse‐free survival between patients who develop ES or those who do not, at least in breast cancer [12]. Another interesting aspect is that our patient had an uncomplicated course after the first transplantation for multiple myeloma, and it is difficult to understand how the second batch of reinfused stem cells from the same collection date made a difference that led to ES. Even though ES has been reported relatively frequently in the medical literature, its rare incidence and variability in signs and symptoms often hinder an early diagnosis. Most biomarkers lack specificity and therefore cannot be diagnostic. For example, a high C‐reactive protein was observed in most cases of ES and seems to be a sensitive marker, although not specific [9]. Another important parameter for early diagnosis and follow‐up is procalcitonin, which is an early reactant in bacterial infections. Our patient presented with negative to low levels of procalcitonin. Knoll et al. has shown that PCT with a cutoff of <  2 ng/mL might be a substantial biomarker in the diagnosis of patients with noninfectious fever associated with ES following ASCT [15]. Early recognition is a key part of successful management of ES as it allows for rapid initiation of a methyl prednisolone therapy. Several authors have suggested a dose of at least 1–1.5 mg/kg of prednisolone or methylprednisolone no later than 48 h after the first episode of fever, followed by a dose tapering of 11–14 days [16]. In the case of refractoriness, a biopsy of affected organs is indicated to confirm the diagnosis. If confirmed, further immunosuppressive therapy can be initiated [17].

## 4. Conclusion

ES is a variable clinical syndrome, which remains underdiagnosed. Due to the complexity of its clinical presentation, it may mimic infectious complications or drug toxicity that may also be associated to the posttransplant phase. While fatal outcomes have been reported, most patients can effectively be treated with immunosuppressive therapy. It is important to raise awareness regarding this rare clinical entity, and the establishment of precise diagnostic criteria and therapeutic regimes is warranted.

## Funding

No funding was received for this manuscript.

## Conflicts of Interest

The authors declare no conflicts of interest.

## Data Availability

The data that support the findings of this study are available on request from the corresponding author. The data are not publicly available due to privacy or ethical restrictions.
